# Effects of Treadmill Exercise on Gut Microbiota in Alzheimer’s Disease Model Mice and Wild-Type Mice

**DOI:** 10.3390/microorganisms13081765

**Published:** 2025-07-29

**Authors:** Zhe Zhao, Xingqing Wu, Wenfeng Liu, Lan Zheng, Changfa Tang

**Affiliations:** Hunan Provincial Key Laboratory of Physical Fitness and Sports Rehabilitation, Hunan Normal University, Changsha 410012, China; zhaoz202506@163.com (Z.Z.); wuxingqing2004@163.com (X.W.); wfliu@hunnu.edu.cn (W.L.)

**Keywords:** Alzheimer’s disease, gut microbiota, treadmill exercise

## Abstract

There is a growing body of research showing that Alzheimer’s disease (AD) is related to enteric dysbacteriosis. Exercise can be effective in alleviating AD, but the effects that exercise has on the gut microbiota in AD patients needs to be further studied. Through this study, we aimed to investigate the differences in the diversity of gut microorganisms between AD model mice and wild-type mice and the effect that treadmill exercise has on the composition of the gut microbiota in both types of mice. C57BL/6 wild-type mice were randomly divided into a sedentary control group (WTC) and an exercise group (WTE); APP/PS1 double transgenic mice were also randomly divided into a sedentary control group (ADC) and an exercise group (ADE). After the control group remained sedentary for 12 weeks and a 12-week treadmill exercise intervention was adopted for the exercise group, the rectal contents were collected so that they could undergo V3-V4 16S rDNA sequencing, and a comparative analysis of the microbial composition and diversity was also performed. The alpha diversity of the gut microbiota in AD mice was lower than that in wild-type mice, but exercise increased the gut microbial diversity in both types of mice. At the phylum level, the dominant microorganisms in all four groups of mice were Bacteroidetes and Firmicutes. There was an increase in the Bacteroidetes phylum in AD mice. Treadmill exercise reduced the abundance of Bacteroidetes in both groups of mice, whereas the abundance of Firmicutes increased. At the genus level, *Muribaculaceae*, the *Lachnospiraceae_NK4A136_group*, *Alloprevotella*, and *Alistipes* were in relatively high abundance. *Muribaculaceae* and *Alloprevotella* were in greater abundance in AD mice than in wild-type mice, but both decreased after treadmill exercise. Through performing linear discriminant analysis effect size (LEfSe), we found that the dominant strains in AD mice were *Campilobacterota*, *Helicobacteraceae*, *Escherichia–Shigella*, and other malignant bacteria, whereas exercise resulted in an increase in probiotics among the dominant strains in both types of mice. Although gut microbial diversity decreases and malignant bacteria increase in AD mice, treadmill exercise can increase gut microbial diversity and lead to the development of dominant strains of probiotics in both types of mice. These findings provide a basis for applying exercise as a treatment for AD.

## 1. Introduction

Alzheimer’s disease (AD) is a progressive multifactorial neurodegenerative disorder that is widely prevalent among elderly individuals. The clinical features of AD are severe deficits in memory, cognition, and motor functions, resulting in a decline in mental, behavioral, and functional activities, which affects the quality of individuals’ daily lives [1]. According to the World Health Organization, nearly 50 million people worldwide suffer from AD. By 2030, 82 million people are expected to be affected by AD, and by 2050, the number of affected individuals is estimated to reach 152 million [2]. Therefore, AD has become a major disease worldwide that is challenging to treat. The pathophysiology of AD is still being explored. In recent decades, several hypotheses have been proposed, among which the amyloid cascade hypothesis and the abnormal tau phosphorylation hypothesis are widely accepted and lead to neuroinflammation, altered calcium homeostasis, vascular degeneration, and ultimately neuronal death [3]. However, the continued failure of clinical drug trials targeting the above pathologic features suggests that it is necessary to reassess the pathogenesis of AD from different points of view.

Various studies have recently shown that there are significant differences in the composition of the gut microbiota between AD patients and healthy controls [4,5]. AD patients have more abundant proinflammatory bacteria and fewer anti-inflammatory bacteria compared with healthy subjects [6]. Morris et al. reported that, in 85% of AD patients, the composition of the gut microbiota was different from that in the healthy population [7]. Compared with normal AD mice, germ-free AD mice have abnormal gastrointestinal motility and decreased amyloid β-protein (Aβ) in the brain, which significantly increases after germ-free mice receive fecal microbiota transplantation (FMT) from normal AD mice, whereas their brain Aβ level shows no obvious change after FMT from wild-type mice [8]. Long-term, broad-spectrum antibiotic therapy can cause enteric dysbacteriosis in AD mice, resulting in significant changes in brain Aβ and glial cells [9]. Therefore, the gut microbiota plays an important role in the occurrence and development of AD and may be a new target for the effective treatment of AD.

A growing body of clinical evidence indicates that exercise, as a nonpharmaceutical method, has a positive effect on the prevention and treatment of cognitive decline in elderly individuals [10,11]. The vibration of the gastrointestinal tract during exercise and the redistribution of blood flow, leading to intestinal hypoxia, may both have an impact on the gut microbiota [12]. However, how exercise influences the gut microbiota of AD patients remains unclear. Through this study, we investigated the differences in the gut microbiota between APP/PS1 transgenic AD mice and wild-type mice and analyzed the effects of treadmill exercise on the composition of the gut microbiota in both types of mice. We aimed to provide an experimental basis for diverse intervention studies on AD.

## 2. Materials and Methods

### 2.1. Animals

APP/PS1 double transgenic mice express a chimeric mouse/human amyloid pre-cursor protein (Mo/HuAPP695swe) and a mutant human presenilin-1 (PS1-dE9) gene, leading to early-onset Alzheimer’s disease; this is currently recognized as an ideal model organism for AD research worldwide [13]. We purchased 12 SPF level, 3-month-old male APP/PS1 double transgenic mice and 12 SPF level, 3-month-old male C57BL/6 mice from Changzhou Cavens Laboratory Animal Co. Ltd. (License No. SCXK (Su) 2016--0010, Changzhou, China). APP/PS1 mice were randomly divided into an AD sedentary control group (ADC) and an AD exercise group (ADE); C57BL/6 mice were also randomly divided into a WT sedentary control group (WTC) and a WT exercise group (WTE). All of the mice were raised in well-ventilated and sealed plastic cages, with free access to food and water, and a circadian environment with 12 h of light and 12 h of darkness was maintained, along with a temperature between 22 °C and 25 °C. These experimental procedures were approved by the Professional Committee of Ethology of Hunan Normal University (approval number: 2022513; approval date: 7 November 2022).

### 2.2. Exercise Intervention Scheme

The exercise intervention was performed using an animal experimental treadmill (Huai Bei Zheng Hua, Huai Bei, Anhui Province, China). The mice in the WTE group and the ADE group underwent treadmill exercise training. In accordance with our previous experimental exercise program [14], the intensity of the exercise gradually increased from the 1st week (at a speed of 7 m/min) to the 8th week (at a speed of 14 m/min), and a constant training speed of 15 m/min was maintained from weeks 9 to 12. Training was conducted 5 days per week for 45 min daily. Moreover, the mice in the WTC and ADC groups were allowed to rest for 12 weeks. When the WTE and ADE groups were subjected to treadmill exercise, the WTC and ADE groups were placed on a stationary treadmill.

### 2.3. Morris Water Maze Test

After the 12-week exercise intervention, the mice were subjected to the Morris water maze test. The procedure followed the method described in our previous study [13], recording the latency to find the platform over the first five days, and the number of crossings over the platform location on the sixth day after the platform was removed, to assess the mice’s learning and memory abilities. Following the six days of behavioral testing, the mice were fasted for 12 h, after which they were deeply anesthetized with isoflurane. The rectal fecal pellets were then dissected and rapidly frozen in liquid nitrogen before being stored at −80 °C for further analysis.

### 2.4. 16S rDNA Sequencing

Total genomic DNA was extracted from the samples using the CTAB/SDS method. The primers 341F (5′-CCTACGGGRBGCASCAG-3′) and 806R (5′-GGACTACNNGGGTATCTAAT-3′) were utilized for the PCR amplification of the V3–V4 variable regions. The mixed PCR products were purified using the Qiagen Gel Extraction Kit (Qiagen, Hilden, Germany). Library construction was performed using the TruSeq^®^ DNA PCR-Free Sample Preparation Kit (Illumina, San Diego, CA, USA), and the NovaSeq 6000 platform (Illumina, USA) was utilized for sequencing.

### 2.5. Statistical Analysis

The sequences were spliced into tags using 16S rDNA sequencing. A series of studies, such as species classification, operational taxonomic unit (OTU) analysis, diversity analysis, and comparative analysis among multiple samples, were subsequently performed on the basis of the tags. For data processing, Mothur was used for the removal of redundant entries from the sequences, and the RDP classifier, which is based on the naive Bayes algorithm, was used for species annotation of the tags. The number of OTUs was calculated at the 97% similarity level, and the OTUs were annotated on the basis of the mode principle. In addition, the Phylogenetic Investigation of Communities by Reconstruction of Unobserved States (PICRUSt) was used to annotate the functional pathways of the OTUs, Mothur was used to analyze the α diversity of the samples, and R was used to conduct the β diversity analysis and draw Venn diagrams. Differences among the four groups were analyzed using a two-way ANOVA via SPSS 21, and these differences were considered statistically significant when the *p*-value was <0.05.

## 3. Results

### 3.1. Sequencing Data

**Figure 1:**
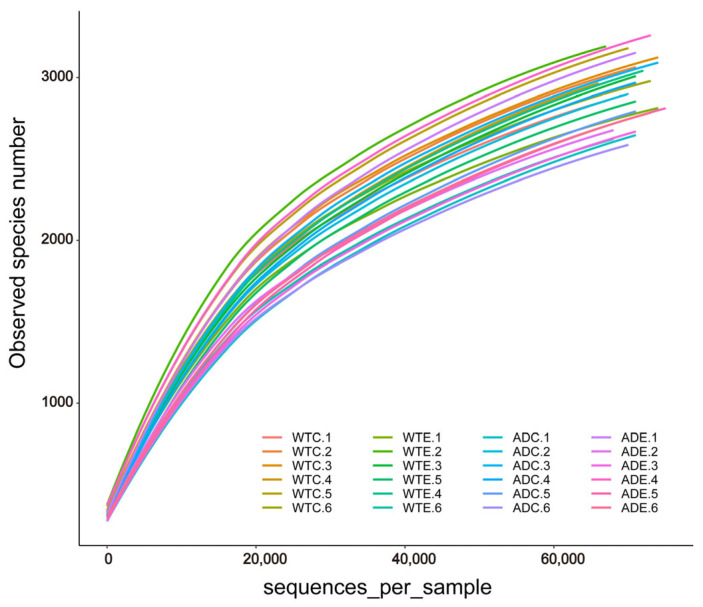
Dilution curves of the gut microbial samples.

**Figure 2:**
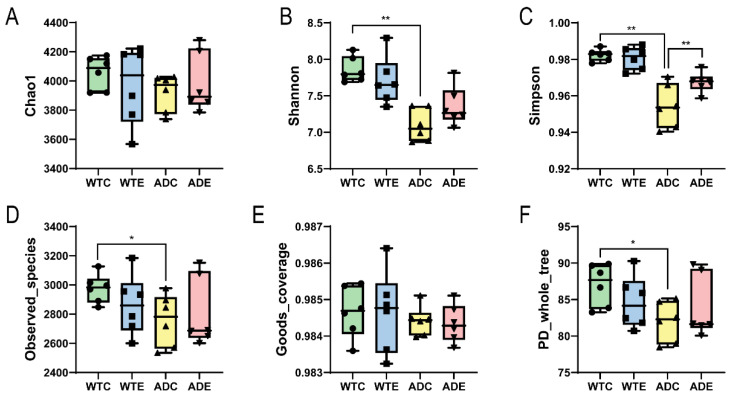
α diversity indices of the gut microbiota. (**A**) Chao1 index; (**B**) Shannon index; (**C**) Simpson index; (**D**) Observed_species index; (**E**) Goods_coverage index; (**F**) PD_whole_tree index. *: *p* < 0.05; **: *p* < 0.01.

After we obtained the sequencing results of the Illumina MiSeq platform, OTU clustering was performed on all sequences according to 97% similarity. Dilution curves were plotted on the basis of the OTU data. As shown in Figure 1, the dilution curves gradually tended to flatten. The addition of more sequencing data generated only a small number of new OTUs, and the sequencing depth coverage index of all the samples was above 98.4% (Figure 2E). These results indicate that the sampling quantity of this experiment was sufficient, and we captured a large proportion of the diversity of the murine gut microbiota.

### 3.2. Analysis of Murine Gut Microbial Diversity

#### 3.2.1. α Diversity Analysis

As shown by the α diversity indices, such as the Chao1 and Simpson indices in Figure 2, WTE > WTC and ADE > ADC, indicating that treadmill exercise can increase the gut microbial diversity of both WT and AD mice. Moreover, WTC > ADC suggests that the gut microbial diversity of AD mice is less than that of WT mice. Compared with the difference in the α diversity indices between the ADE and ADC groups, the difference between the WTE and WTC groups was smaller, suggesting that AD mice were more sensitive to treadmill exercise. These results indicate that the gut microbial diversity of AD mice is lower than that of wild-type mice, whereas the improvement is more significant after treadmill exercise.

#### 3.2.2. β Diversity Analysis

**Figure 3:**
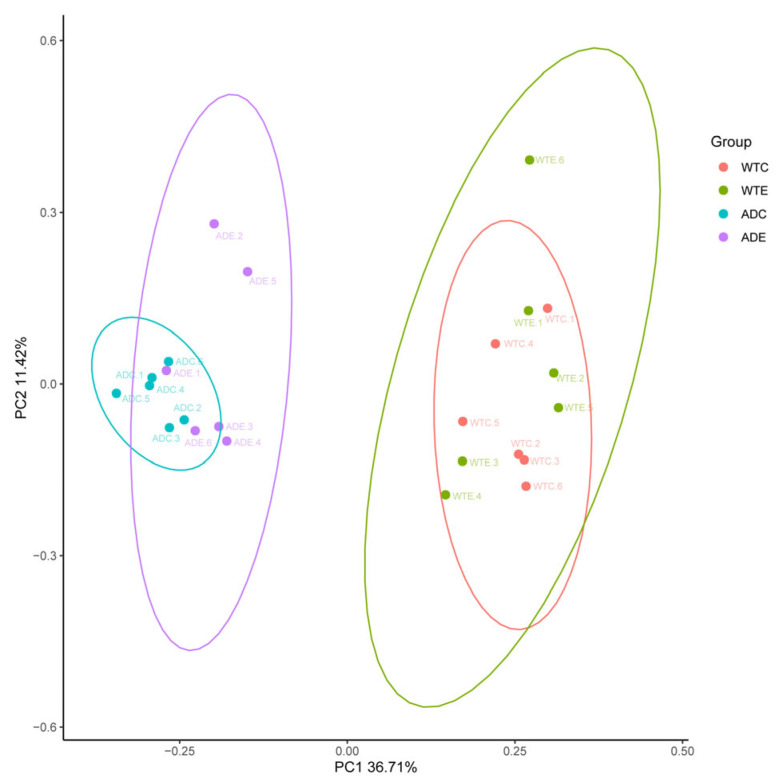
Principal coordinate analysis (PCoA).

As shown in Figure 3, the samples from the WTC and ADC groups were relatively concentrated and showed intragroup aggregation more obviously. The samples from the WTE and ADE groups were more dispersed, and the intragroup disparity increased. These findings indicate that treadmill exercise can increase interindividual differences in the murine gut microbiota.

### 3.3. Analysis of the Composition of the Murine Gut Microbiota

#### 3.3.1. Venn Diagram Analysis of the Species

**Figure 4:**
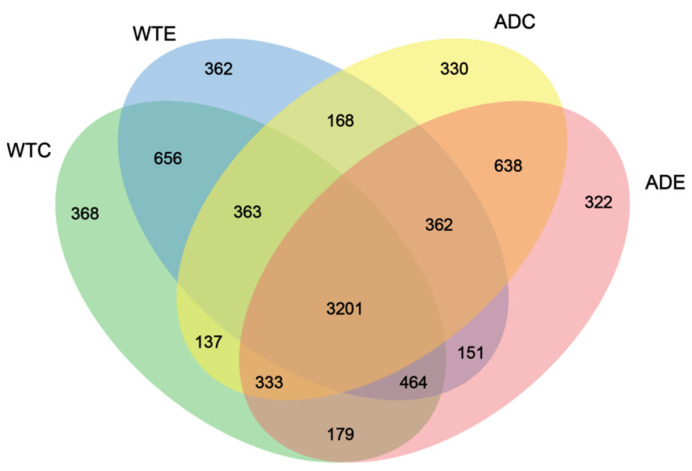
Venn diagram of the murine gut microbiota.

As shown in Figure 4, 3201 OTUs were common to the four groups of mice, accounting for the vast majority of the total. The WTC group had the largest number of unique OTUs, at 368, whereas the ADE group had the smallest number, at 322. There were 362 unique OTUs in the WTE group and 330 unique OTUs in the ADC group, suggesting that the number of unique species of gut microbiota in AD mice was lower than that in WT mice and that treadmill exercise played a role in reducing the number of unique species of gut microbiota in both types of mice. In addition, the number of OTUs shared by the WTC and ADC groups was 4034 (363 + 3201 + 333 + 137), whereas that of the WTC and ADE groups was 4177 (333 + 3201 + 464 + 179). The above results indicate that treadmill exercise can change the composition of the murine gut microbiota, reduce the number of unique species, and increase the similarity of the gut microbes of AD mice to those of normal mice.

#### 3.3.2. Species Composition of the Murine Gut Microbiota at the Phylum Level

**Figure 5:**
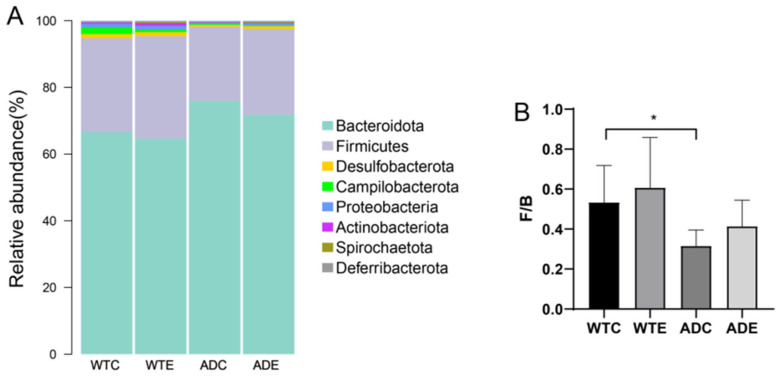
Species composition of the murine gut microbiota at the phylum level. (**A**) Stacked histograms of the murine gut microbiota at the phylum level; (**B**) Firmicutes/Bacteroidetes ratio (F/B). *: *p* < 0.05.

At the phylum level, Bacteroidetes and Firmicutes were the dominant microorganisms in all four groups of mice. Bacteroidetes was more abundant in AD mice. Treadmill exercise reduced the abundance of Bacteroidetes in both types of mice, whereas the abundance of Firmicutes increased (Figure 5A). To present the differences in proportions between the two phyla more intuitively, the Firmicutes-to-Bacteroidetes ratios (F/B) were analyzed (Figure 5B). Compared with that of WT mice, the F/B ratio of AD mice was significantly lower. Treadmill exercise increased the F/B ratios of both types of mice, but the differences were not significant.

#### 3.3.3. Species Composition of the Murine Gut Microbiota at the Genus Level

**Figure 6:**
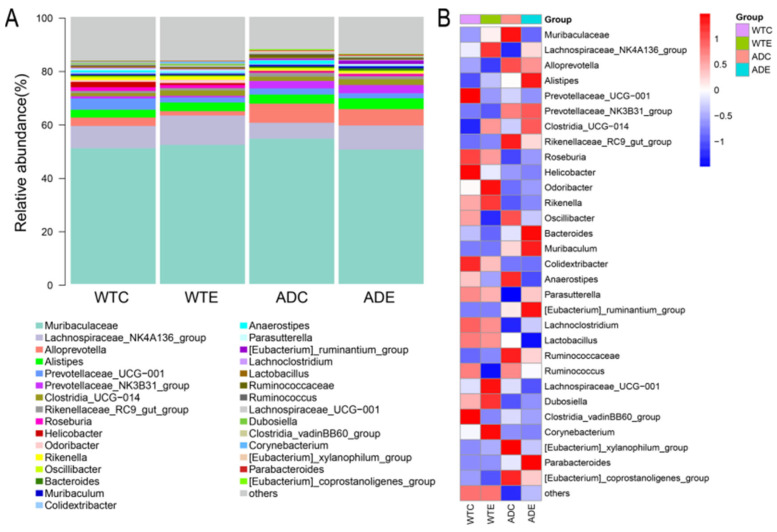
Species composition of the murine gut microbiota at the genus level. (**A**) Stacked histograms of the murine gut microbiota at the genus level; (**B**) heatmap of the murine gut microbiota at the genus level.

As shown in Figure 6, at the genus level, *Muribaculaceae*, the *Lachnospirace-ae_NK4A136_group*, *Alloprevotella*, and *Alistipes* accounted for a large proportion of the total number of genera, indicating that their relative abundance was high. The abundance of *Muribaculaceae* and *Alloprevotella* in AD mice was greater than that in wild-type mice, and both decreased after treadmill exercise was performed. However, the *Lachnospiraceae_NK4A136_group* was less abundant than in wild-type mice, but this improved after treadmill exercise was performed.

### 3.4. LEfSe Analysis of Murine Gut Microbiota

**Figure 7:**
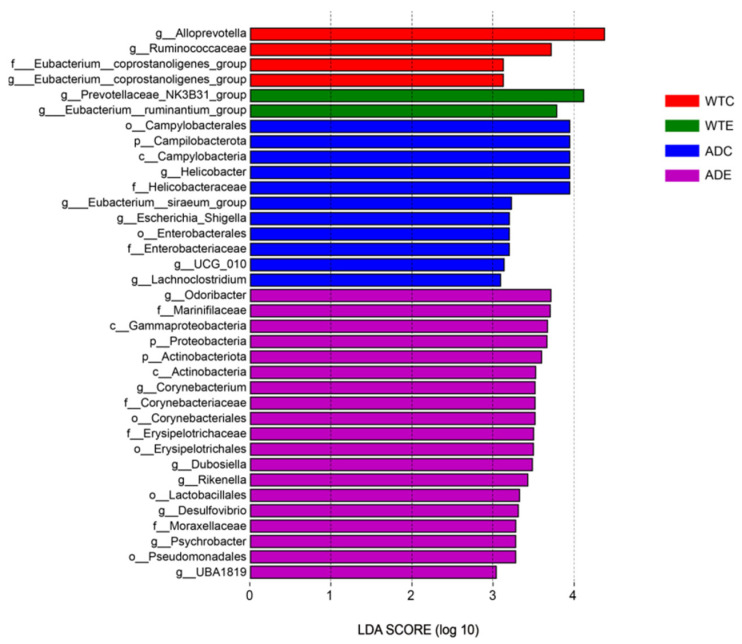
LEfSe analysis.

We performed LEfSe analysis of the gut microbiota of the four groups of mice, and the LDA score was set to 4.0. As shown in Figure 7, the dominant strains of the WTC group were *Alloprevotella*, *Ruminococcaceae*, and the *Eubacterium coprostanoligenes group*; the dominant strains of the WTE group were the *Prevotellaceae NK3B31 group* and the *Eubacterium ruminantium group*; the dominant strains of the ADC group were *Campilobacterota*, *Campylobacteria*, *Enterobacterales*, *Helicobacteraceae*, *UCG 010*, the *Eubacterium siraeum group*, *Escherichia–Shigella*, and *Lachnoclostridium*; and the dominant strains of the ADE group were *Proteobacteria*, *Actinobacteriota*, *Gammaproteobacteria*, *Corynebacteriales*, *Erysipelotrichales*, *Lactobacillales*, *Pseudomonadales*, *Marinifilaceae*, *Corynebacteriaceae*, and *Odoribacter*.

### 3.5. Random Forest Analysis of the Murine Gut Microbiota

**Figure 8:**
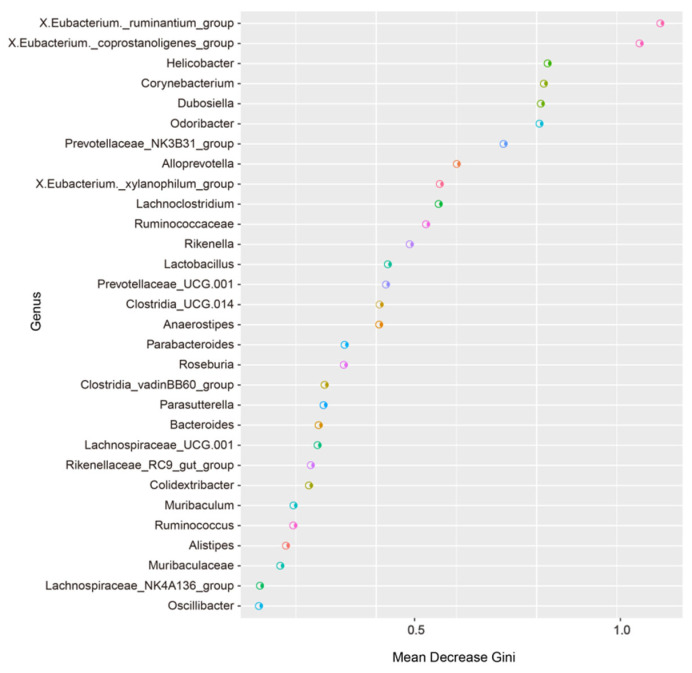
Random forest analysis.

The random forest importance plot showcases the key species, reflecting the differences between groups. As shown in Figure 8, the key species were the *Eubacterium ruminantium group*, the *Eubacterium coprostanoligenes group*, *Helicobacter*, *Corynebacterium*, *Dubosiella*, *Odoribacter*, the *Prevotellaceae NK3B31 group*, *Alloprevotella*, *X.Eubacterium*, the *_xylanophilum_group*, *Lachnoclostridium*, *Ruminococcaceae*, and *Rikenella*.

### 3.6. Gene Function Analysis of the Murine Gut Microbiota

**Figure 9:**
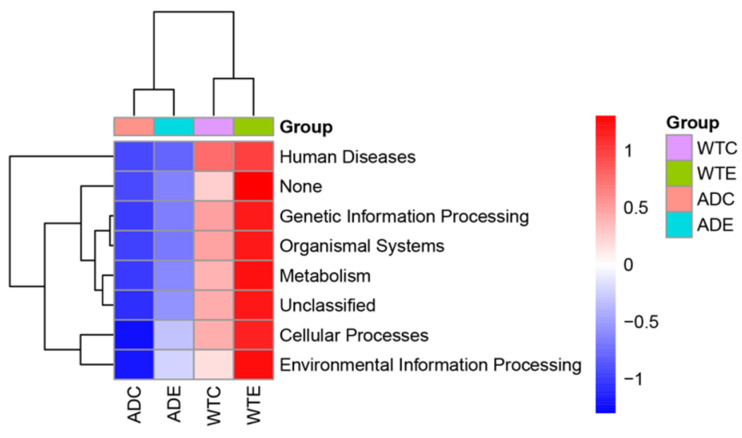
Predictive functional profiling by PICRUSt.

With the aid of PICRUSt, we predicted the composition of known microbial gene functions and analyzed the functional differences among the four groups. We subsequently performed an analysis of these functional differences on the basis of the predicted KEGG results at level 1. As shown in Figure 9, the expression of KEGG pathways such as human diseases, genetic information processing, organismal systems, metabolism, cellular processes, and environmental information processing in the ADC group was generally low, though it was upregulated after exercise intervention took place. However, the expression of the KEGG pathways was relatively high in the WTC group, and this further increased after the exercise intervention took place. These results indicate that AD mice have a lower expression of gut microbial functions and that exercise may influence the host’s health by affecting the gut microbiota and thereby the signaling pathways mentioned above.

### 3.7. Correlation Analysis Between Gut Microbiota and Morris Water Maze Performance in Mice

**Figure 10:**
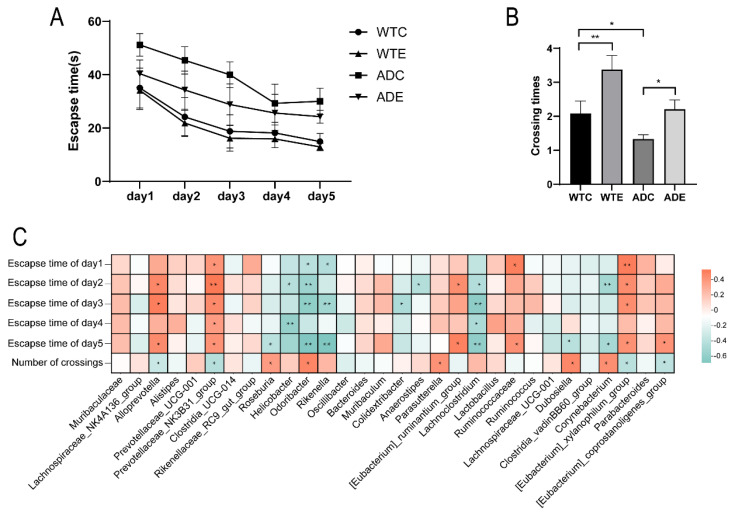
Correlation analysis between gut microbiota and Morris water maze performance in mice. (**A**) Latency during the Morris water maze test in mice. (**B**) Number of platform crossings during the Morris water maze test in mice. (**C**) Spearman correlation heatmap between gut microbiota and Morris water maze performance in mice. *: *p* < 0.05; **: *p* < 0.01.

The Morris water maze test showed that the latency of AD mice was higher than that of wild-type mice during the first five days, with a reduction in latency following treadmill exercise (Figure 10A). After platform removal, AD mice exhibited a significantly reduced number of crossings over the platform location compared to wild-type mice, which significantly increased after treadmill exercise (Figure 10B). These results indicate that treadmill exercise can significantly improve the learning and memory abilities of AD mice. Spearman correlation analysis between the gut microbiota and Morris water maze performance revealed that *Alloprevotella*, the *Prevotellaceae_NK3B31_group*, the *[Eubacterium]_xylanophilum_group*, and others were significantly positively correlated with latency and significantly negatively correlated with the number of platform crossings. In contrast, *Odoribacter*, *Rikenella*, *Corynebacterium*, and others were significantly negatively correlated with latency and significantly positively correlated with the number of platform crossings (Figure 10C). These data suggest a strong association between the gut microbiota and Morris water maze performance, and exercise may regulate the mice’s learning and memory abilities by modulating the gut microbiota.

## 4. Discussion

Humans have trillions of symbiotic microorganisms, including bacteria, archaea, viruses, and eukaryotic microorganisms. These microorganisms exist both inside and outside the body, among which the colon is the most abundant and important site for colonization [15]. An increasing number of studies have shown that the gut microbiota can control the functions of the central nervous system through the gut—brain axis and that changes in the composition and functions of the microbial community can promote the development and progression of AD [16]. The diversity of the gut microbiota in AD patients is significantly reduced, especially that of *Bifidobacterium*, *Bacteroides*, *Eubacterium*, and *Parabacteroides* [17]. Through our results generated from this study, we revealed that the total number of OTUs in the gut microbiota of AD mice was the smallest, and the number of unique species decreased. However, exercise played a role in reducing the unique species of both WT and AD mice. Through further analysis of the α diversity of the murine gut microbiota, we revealed that indices such as the Chao1 estimator and Simpson index were lower in AD mice than in WT mice, whereas treadmill exercise increased the above indices in both types of mice. Zhang et al. reported that the diversity of the gut microbiota in 5- to 6-month-old AD mice was significantly lower than that in littermate controls and that the composition of the microbial community changed significantly with age at the phylum, genus, and species levels [18]. Varghese et al. summarized over 100 studies on exercise and the gut microbiota and reported that a moderate amount of exercise can increase the diversity of the gut microbiota [19]. These findings suggest that the results of our study are consistent with those of the current research landscape. The diversity of the gut microbiota decreased in AD mice, though this improved after the exercise intervention took place. Ling et al. conducted tests on the gut microbiota of 100 Chinese AD patients and 71 age- and sex-matched controls with normal cognition and reported that the microbial community structure of AD patients changed significantly, with a decrease in the α diversity and altered β diversity indices [20]. These results indicate that AD mice, similar to AD patients, exhibit a reduction in gut microbial diversity and that exercise can effectively increase this diversity.

Vogt et al. [21] reported that the abundance, diversity, and unique composition of the gut microbiota in AD patients decreased compared with those in asymptomatic age- and sex-matched controls. The abundance of *Firmicutes* and *Actinobacteria* was significantly reduced, whereas that of *Bacteroidetes* was enriched in AD patients. In this study, *Bacteroidetes* and *Firmicutes* accounted for the vast majority of the bacteria in the four groups of mice at the phylum level. The abundance of *Bacteroidetes* decreased in the WTE group and increased in the ADC group compared with that in the WTC group, which is consistent with the changes in the gut microbiota in AD patients. The abundance of *Bacteroidetes* was lower in the ADE group compared to that in the ADC group, suggesting that exercise can reduce *Bacteroidetes* abundance. Furthermore, *Firmicutes* increased in the WTE group and decreased in the ADC group compared with the WTC group. The abundance of *Firmicutes* in the ADE group was greater than that in the ADC group, indicating that exercise can increase the abundance of *Firmicutes* in the intestine. Various studies have shown that the abundance of *Firmicutes* in the gut microbiota is related to obesity and metabolic disorders [22]. The *Firmicutes*-to-*Bacteroidetes* ratio (F/B) is considered an indicator of intestinal flora imbalance and is associated with diseases such as obesity, inflammatory bowel disease (IBD), and type 2 diabetes [23], which suggests that the overgrowth of *Bacteroidetes* and excessive reduction in *Firmicutes* may be critical factors contributing to the onset of AD. Treadmill exercise could alleviate this disease by decreasing the abundance of *Bacteroidetes* and increasing the abundance of *Firmicutes* in the intestine.

At the genus level, *Muribaculaceae* constituted the predominant proportion of the gut microbiota across all four groups of mice, with an increase in AD mice and a subsequent decrease following exercise intervention. *Muribaculaceae* can generate short-chain fatty acids (SCFAs) through endogenous (mucinoglycans) and exogenous polysaccharides (dietary fiber); thus, *Muribaculaceae* is considered a potential probiotic that can promote host health through a variety of mechanisms [24]. However, *Muribaculaceae* in AD mice was increased, and Zhang et al. [25] reported that the Jiedu Yizhi formula could regulate enteric dysbacteriosis in AD mice by reducing the abundance of *Muribaculaceae*, thereby improving cognitive function. Therefore, the role that *Muribaculaceae* plays in AD remains to be confirmed. In addition, exercise can increase the abundance of microorganisms such as the *Lachnospiraceae_NK4A136_group* and *Clostridia-UCG-014* in both WT and AD mice and reduce the abundance of *Alloprevotella* and *Prevotellaceae UCG-001*. The *Lachnospiraceae_NK4A136_group* can ferment dietary fiber and mucoprotein to produce SCFAs; these metabolites are crucial for intestinal health [26]. However, the abundance of this genus is relatively low in AD mice, and its levels are closely associated with overall health status [27]. Moreover, An et al. reported that an increase in the *Lachnospiraceae_NK4A136_group* was correlated with a reduced risk of AD [28]. Therefore, the decrease in the abundance of the *Lachnospiraceae_NK4A136_group* may lead to the aggravation of AD pathology, whereas exercise may mitigate AD-related pathological changes by increasing the abundance of this genus. *Clostridia UCG-014* is an anaerobic bacterium capable of fermenting dietary fibers to produce SCFAs such as propionic acid and butyric acid, which, in some cases, exhibits proinflammatory properties, but its potential to produce SCFAs and modulate intestinal health makes it a potential probiotic [29]. Therefore, exercise may improve AD by regulating the abundance of the *Lachnospiraceae_NK4A136_group* and *Clostridia UCG-014*. *Alloprevotella*, associated with oral diseases such as periodontitis, is associated with the risk of cardiovascular diseases in the intestine and is present mainly in the oral cavity and intestinal tract of the human body [30]. *Prevotellaceae UCG-001* plays a positive role in regulating the structure and function of the gut microbiota, but it is also correlated with a variety of metabolic disorders. In hyperglycemic mouse models, the abundance of this genus significantly increases, and it is related to impaired glucose regulation (IGR) and lipid metabolism disorders [31]. Consequently, exercise may enhance health outcomes by reducing the abundance of *Prevotellaceae UCG-001* and *Alloprevotella* within the murine gut microbiota.

Through LEfSe analysis, we revealed that the dominant bacteria in the WTE group of mice were the *Prevotellaceae NK3B31 group* and the *Eubacterium ruminantium group*. The *Prevotellaceae NK3B31 group* can produce SCFAs, such as acetic acid, propionic acid, and butyric acid, which play key roles in maintaining intestinal health and regulating immune responses [32]. The *Eubacterium ruminantium group* helps to maintain the integrity of the intestinal mucosal barrier, reduce inflammation levels, and regulate the immune response by generating SCFAs (especially butyric acid). A decrease in its abundance may be associated with metabolic diseases such as obesity and type 2 diabetes [33]. Among the dominant bacteria in the ADC group of mice, *Campilobacterota* is a bacterial phylum containing multiple important pathogens and is one of the main causes of bacterial gastroenteritis worldwide [34]. *Helicobacter* is an important genus of *Helicobacteraceae*, among which the most famous is *Helicobacter pylori*, a notable pathogen that can cause gastric diseases such as chronic gastritis, peptic ulcers, gastric adenocarcinoma, and low-grade gastric lymphoma [35]. The *Eubacterium siraeum group* is a group of bacteria that can ferment carbohydrates to produce SCFAs (such as butyric acid and acetic acid), which play important roles in maintaining the intestinal health and metabolic regulation of the host. However, in some cases, *Eubacterium siraeum* can cause mixed infection with other anaerobes or facultative anaerobes, leading to diseases such as endocarditis [36]. *Escherichia–Shigella* is a bacterial community that includes the genera *Escherichia* and *Shigella*, which belong to *Enterobacteriaceae*. *Shigella* comprises the main pathogens causing bacillary dysentery, and some *Escherichia* strains can also cause similar intestinal infections [37,38]. Various studies have shown that the abundance of *Escherichia–Shigella* is significantly increased in patients with sarcopenia [39]. The above evidence indicates that *Escherichia–Shigella* bacteria are harmful. Many studies have shown that the bacteria of *Lachnoclostridium* are malignant and closely related to the occurrence of metabolic diseases such as obesity, hypertension, and diabetes [40,41]. A Mendelian randomization study indicated a causal relationship between *Lachnoclostridium* and AD [42]. Ubeda et al. reported that *Lachnoclostridium* increased in the feces of AD patients [43]. Therefore, the overgrowth of harmful bacteria such as *Campilobacterota*, the *Eubacterium siraeum group*, *Escherichia–Shigella*, and *Lachnoclostridium* in AD mice may be notable for accelerating AD pathology. Among the dominant bacteria in the ADE group of mice, *Odoribacter* is a common member of the human gut microbiota and participates in intestinal metabolic processes, especially the generation of SCFAs. A decrease in the abundance of *Odoribacter* is correlated with a variety of diseases, including IBD, nonalcoholic fatty liver disease (NAFLD), and cystic fibrosis [44]. Studies have shown that *Odoribacter laneus* can improve the metabolic status of obese mice by consuming succinic acid, demonstrating its potential as a next-generation probiotic [45]. *Dubosiella newyorkensis* has probiotic immunomodulatory effects and can regulate immune responses by generating SCFAs (such as propionic acid) and L-lysine [46]. *Dubosiella newyorkensis* can improve cognitive impairments and inhibit neuroinflammation by regulating the structure of the intestinal flora and metabolites, but it has no toxic effects on major organs. However, its abundance was significantly reduced in AD mice [47]. Furthermore, it has also been found to exhibit antiaging effects in aged mice [48]. *Dubosiella newyorkensis* is considered a potential therapeutic target, especially in the treatment of diseases such as IBD and AD [49]. Studies in recent years have shown that *Rikenella* plays a significant role in maintaining metabolic homeostasis, the immune system, and intestinal health, and its abundance is closely related to the host immune system, making it an anaerobic bacterium with potential probiotic functions [50,51]. Xiao et al. reported that *Desulfovibrio* inhibits the production of glucagon-like peptides in the intestine by generating hydrogen sulfide, thereby affecting the metabolic balance of the host and promoting the occurrence of metabolic diseases such as obesity [52], which indicates that *Desulfovibrio* may be harmful bacteria. *UBA1819* can ferment carbohydrates to produce SCFAs. Its abundance is positively correlated with the level of uracil in serum, and uracil plays an important role in nerve regeneration and anti-inflammatory processes [53]. This evidence indicates that exercise can increase the abundance of potential probiotics such as *Odoribacter*, *Dubosiella*, *Rikenella*, and *UBA1819* in AD mice, as well as the abundance of the potentially harmful bacteria *Desulfovibrio*, suggesting that exercise is more likely to promote the development of the gut microbiota in both types of mice in the direction of probiotics.

We further screened the potential biomarkers using random forest analysis, and we revealed that the key species reflecting the differences among the four groups of mice were the *Eubacterium ruminantium group*, the *Eubacterium coprostanoligenes group*, *Helicobacter*, *Corynebacterium*, *Dubosiella*, and *Odoribacter*. The results were similar to those of the LEfSe analysis, indicating that these bacteria play important roles in the gut microbiota of the two types of mice. However, how the abovementioned bacteria affect the health status of AD patients needs to be explored further. In predicting the composition of known microbial gene functions, AD mice present low expression of genes associated with KEGG pathways such as human diseases, genetic information processing, organismal systems, metabolism, cellular processes, and environmental information processing. Exercise upregulated the above pathways in both types of mice, indicating that the microbial community metabolism in AD mice is relatively weak, and that exercise can improve the metabolism in both types of mice. Exercise may affect host health by regulating the gut microbiota and thus through the abovementioned relevant pathways; however, further research into how exercise interacts with metabolic pathways is needed.

In conclusion, the diversity of the gut microbiota in AD mice declines, with an increase in harmful bacteria. Treadmill exercise can increase the diversity of gut microbes in both types of mice and promote the development of dominant strains of probiotics. These findings elucidate the composition of and differences in the gut microbiota between these two types of mice and demonstrate the effects of exercise on their intestinal microbial communities. These findings establish a foundation for the future screening of beneficial microorganisms and the treatment of AD patients through diverse modulations of the gut microbiota, as well as offer novel evidence to explain the health-promoting effects of exercise from the perspective of gut microbiota.

## Data Availability

The original contributions presented in this study are included in the article. Further inquiries can be directed to the corresponding authors.
